# “It's just us”: Families' experiences with temporary tube feeding

**DOI:** 10.1002/ncp.70019

**Published:** 2025-08-20

**Authors:** Claire Reilly, Jeanne Marshall, Jasmine Foley, Nikhil Thapar, Rebecca Packer

**Affiliations:** ^1^ School of Health and Rehabilitation Sciences The University of Queensland Brisbane Queensland Australia; ^2^ Queensland Children's Hospital, Children's Health Queensland Hospital and Health Service South Brisbane Queensland Australia; ^3^ Gastroenterology, Hepatology and Liver Transplant Queensland Children's Hospital (QCH) Brisbane Queensland Australia; ^4^ Faculty of Health, School of Exercise and Nutrition Sciences Queensland University of Technology Brisbane Queensland Australia; ^5^ School of Medicine The University of Queensland Brisbane Queensland Australia

**Keywords:** enteral feeding, parents, patient education, pediatrics, qualitative research

## Abstract

**Background:**

Children with temporary feeding tubes are discharged home with increasing frequency, yet little is known about how families adapt and manage in their home environment. Whereas the physical side effects of temporary feeding tubes are well documented, the psychosocial impact on families remains underresearched. Understanding families' evolving needs is critical to improving care and reducing caregiver burden.

**Aim:**

To explore parents' experiences of caring for children with temporary feeding tubes, from insertion to removal and to identify their challenges and evolving needs.

**Methods:**

A longitudinal qualitative descriptive design was used. Parent participants completed diaries and semistructured interviews across three key time points in their child's tube feeding journey (initial, maintenance, final) over a 4‐month period. Inductive content analysis was used to analyze data.

**Results:**

Thirty‐six parent participants completed 81 interviews and 223 diary entries, documenting their experiences over time. An integrative theme identified was families' critical need for ongoing support. Parents were unprepared for tube feeding and faced persistent challenges managing the feeding tube. Their journey transformed from initial uncertainty to self‐taught expertise, as they adapted to changing demands. Their need for professional and peer support remained constant throughout.

**Conclusion:**

These findings underscore the need for systematic improvements, including structured education, consistent follow‐up, accessible clinical expertise, and support across the tube feeding journey. Addressing these gaps could improve family well‐being, reduce healthcare inequities, and enhance clinical outcomes.

## BACKGROUND

Temporary feeding tubes such as nasogastric, orogastric, or postpyloric tubes are commonly used to provide short‐term nutrition support to children across a wide range of medical conditions, throughout all stages of treatment and recovery.^1^, ^2^, ^3^, ^4^ Up to 25% of children may require a temporary feeding tube during hospital admission, with 17% of these potentially being discharged home with one in place.^4^, ^5^ Advances in technology^6^ and the global shift from hospital to home care, aiming to reduce length of hospital stay, have contributed to an increasing trend of discharging children home with temporary feeding tubes.^7^, ^8^, ^9^, ^10^ With growing numbers of children at home with temporary feeding tubes, health services and clinicians must understand how these tubes are managed in the home setting^11^, ^12^, ^13^ and how they impact family functioning and well‐being.^14^

The physical side effects of temporary feeding tubes, including vomiting, gagging, and skin irritation,^15^, ^16^ are well documented. However, literature exploring the psychosocial impact of temporary feeding tubes on families remains relatively limited. Existing studies indicate that parenting a child with a feeding tube is emotionally challenging, exhausting, and stressful,^17^, ^18^, ^19^, ^20^, ^21^, ^22^ with substantial impacts on family life^23^ and increased risks of maternal clinical depression.^24^ Parents also struggle with navigating public perceptions of their child's tube.^25^ This research provides valuable insights, but most studies have included a mixed cohort of children using long‐term feeding tubes (eg, gastrostomy tubes) alongside children using temporary tubes in the participating cohorts.^26^, ^27^, ^28^, ^29^, ^30^, ^31^, ^32^, ^33^, ^34^ As a result, the applicability of these findings to families of children with temporary feeding tubes alone requires further investigation.

Three studies examining families' experiences with temporary feeding tubes reveal consistent patterns of significant caregiver burden, including physical, psychological, and financial challenges.^35^, ^36^, ^37^ Families reported difficulties with tube management, particularly with nasogastric tube replacement, causing considerable stress^37^ and substantial impacts on family life, with parents striving to return to normality.^36^ Although these studies provide valuable insights through their different contexts (hospital discharge, neonatal intensive care unit with remote monitoring, and cardiac care), their limitations include small sample sizes (13–15 participants) and single‐time‐point designs. These constraints reduce the ability to capture how families' experiences evolve throughout the temporary tube feeding journey, highlighting the critical need for larger, more diverse longitudinal research examining families' experiences from tube insertion through removal across different populations, geographical locations, and healthcare settings. This study aimed to explore parents' experiences of caring for children with temporary feeding tubes, from insertion to tube removal, to identify their challenges and evolving needs over time.

## METHODS

### Study design

This study used a longitudinal qualitative descriptive approach to explore the experiences of parents of children with temporary feeding tubes from tube insertion to tube removal over a 4‐month time frame. Four months was selected as the study time frame because this represented the typical duration a child had a temporary feeding tube in place in this hospital.^5^ A qualitative descriptive design using a constructivist approach was selected to capture participants' experiences.^38^, ^39^ Repeated interviews were conducted to understand how parents' experiences evolved over time.^40^ The constructivist approach shaped the study design, data collection, and analysis. Health consumers with lived experience of having a child with a feeding tube reviewed and revised the study design, participant information forms, and interview questions, ensuring the research was grounded in participant perspectives. This study adhered to the Consolidated Criteria for Reporting Qualitative Research (COREQ) 32‐item checklist to ensure comprehensive and transparent reporting of methodology.^41^

### Ethical approval

This study received full ethics approval from the Children's Health Queensland Hospital and Health Service Human Research Ethics Committee (HREC/18/QRCH/168) and from The University of Queensland Human Research Ethics and Integrity Committee (2022/HE000953). Both committees serve as institutional review boards under Australian regulations.

### Participants and sampling

Purposeful sampling was used to ensure representation of different child ages, diagnoses, and geographical locations, capturing a range of perspectives.^42^ Participants were recruited from a large quaternary public children's teaching hospital (see the process of recruitment in Figure S1). Temporary feeding tubes were categorized as nasogastric, nasoduodenal, or nasojejunal tubes. Eligible criteria included children discharged home with a temporary feeding tube; feeding tube used for nutrition/hydration (not solely for medication); and parents aged >18 years and residing in Australia. Because this study was focused on children who required tube feeding to improve nutrition for growth, children with diagnosed eating disorders, those requiring tubes solely for medication, and children with terminal or uncertain prognosis (eg, palliative care) were excluded. Non‐English‐speaking families and children in foster care without legal guardian consent were also excluded.

### Study procedure

Data were collected at three time points: tube insertion, maintenance (typically 1–2 weeks after discharge home), and 4 months after discharge or upon tube removal. For children whose feeding tubes were in place for >1 month at the time of recruitment (*n* = 30), only initial and final interviews were conducted. Data from the initial interview for these participants were grouped with the maintenance phase data to align with the longitudinal design and ensure consistency with the time frame of other participants. Data collection included information from medical records, diaries, and semistructured interviews. Medical records were reviewed by the primary investigator to gather information such as the child's diagnosis, temporary feeding tube details, and home address for determining geographical location. The Australian Statistical Geography Standard Remoteness Structure identified participants' urban or rural home address locations.^43^

Interview and diary questions were developed from the literature and clinical experience (described in Supporting Information S1: Files S1 and S2). Interview questions were designed to facilitate open communication through a flexible semistructured format. Diaries were hosted on an online secure platform (Qualtrics), allowing parents to document experiences asynchronously throughout the study duration. Diary questions were designed to be brief, requiring <5 min for parents to complete. Participants kept diaries to enable them to express their experiences in their own words and at their own pace while minimizing researcher influence. The use of diaries complemented the longitudinal study design by capturing real‐time reflections, a method that has been shown to enhance the richness and authenticity of qualitative data in healthcare research.^44^

Regular follow‐ups, including weekly reminders via phone, email, or SMS, were used to maintain participant engagement in diary completion, consistent with previous studies demonstrating the benefits of ongoing contact in longitudinal research.^45^ Semistructured interviews were conducted by the primary investigator either face‐to‐face or by telephone, based on participant preference. Similar to previous longitudinal studies, interviews were spaced at intervals to capture the progression of participants' experiences over time.^46^ Interviews were audio‐recorded and transcribed verbatim by researchers CR and RP. Member checking was conducted by sharing all transcribed interviews with participants to verify accuracy and allowing participants to verify the data.^47^ Diary entries were retrieved from the online platform (Qualtrics) for analysis.

### Data analysis

Diary entries completed up to the date of the maintenance interview were grouped with initial interview data, whereas diary entries after the maintenance interview were included with maintenance data. Final interviews had no associated diary entries. Qualitative content analysis of interview transcripts and diary entries followed the Graneheim and Lundman approach,^48^ beginning with reading and familiarization by the primary investigator to understand the context and gain familiarity with the content. NVivo version 14 (Lumivero) was used for data organization. Units of meaning were identified, condensed, and coded in an iterative process with team discussions to refine codes. Codes were grouped into subcategories and then into broader categories. Analysis was initially inductive to allow themes to be identified and then became more deductive to align with a constructivist lens, ensuring interpretation was informed by theoretical insights^49^ while remaining grounded in participants' experiences. Themes were formulated across the entire dataset. Triangulation of interviews, diary data, and medical records ensured a comprehensive analysis. To ensure trustworthiness, initial codes generated by the primary investigator were peer‐reviewed in team meetings, with regular peer debriefings and diverse viewpoints, which helped moderate potential biases. The research team included clinicians and researchers with expertise in pediatric nutrition, feeding, and qualitative health research. The primary investigator conducted all interviews and led the analysis with regular team discussions to ensure reflexivity and interpretive rigor.

## RESULTS

Thirty‐six parents of 37 children with temporary feeding tubes, including one set of twins, participated in the study. Nine parent participants were lost to follow‐up, two withdrew voluntarily because of time commitments, and seven were uncontactable within the study time frame (described in recruitment Figure S1). Participant characteristics are presented in Table 1 (parent participants) and Table 2 (child participants). Most primary caregivers identified as mothers (*n* = 31 of 34, 91%), and three identified as fathers (*n* = 3 of 34, 9%). Five families (15%) identified as First Nations, and six families (18%) had a culturally and linguistically diverse background. Geographically, most families lived in metropolitan areas (*n* = 26 of 34, 76%), with six (18%) in regional areas and one (3%) in a remote area. Parents completed 223 diary entries over the study time period (205 by mothers, eight by fathers, and 10 by both parents). A total of 81 interviews (including 16 with fathers) were conducted across three phases: 29 at tube insertion, 24 during maintenance, and 28 at the final time point, with interview durations averaging 8.8, 14.9, and 19.0 min, respectively (SD = 3.7, 4.5, and 10.1, respectively).

**Table 1:** Parent characteristics.

Category	*N* (%) (*N* = 34)
Main caregiver	
Female	31 (91)
Male	3 (9)
Main caregiver age, mean (range), years	34 (23–45)
Working status of participating parent	
Employed/working	8 (24)
On leave	10 (29)
Not working	16 (47)
Education	
Secondary education	11 (32)
Vocational/technical education	9 (26)
Higher education	14 (41)
Family structure	
Siblings at home	22 (65)
Single parent	2 (6)
Household size, mean (median)	4.4 (4)

**Table 2:** Child characteristics.

Category	*N* (*N* = 37)
Sex	
Male	19
Female	18
Age at tube insertion, mean (median), months	30 (3)
Diverse cultural background^a^	
Torres Strait Islander	1
Aboriginal	4
Culturally and linguistically diverse	6
Geographical location	
Metropolitan	26
Regional	10
Remote	1
Tube insertion location	
Queensland Children's Hospital	25
Another hospital	12
Had been at home with the feeding tube before the study	
Yes	5
No	32
Duration child had feeding tube before study enrollment, mean (median), days	60.2 (18)
Tube type	
Nasogastric	36
Transpyloric	1
Tube feeding regimen	
Bolus	22
Continuous	8
Combination of continuous and bolus	7
Tube use at the end of the study	
Tube remained in place	15
Tube was removed	13
Medical teams responsible for tube insertion	
Cardiology	8
General Paediatrics	10
Neonatology	3
Oncology	8
Respiratory	3
Other (Cleft, Metabolic Medicine, Nephrology, Neurology, Rheumatology)	5
Number of medical teams involved with each child, mean (median)	2.5 (2)
Reason for tube insertion	
Nutrition/growth	28
Unsafe swallow/nothing by mouth	9
*ICD‐11* diagnosis	
Certain conditions originating in the perinatal period	5
Congenital malformations, deformations and chromosomal abnormalities	2
Diseases of the circulatory system	8
Diseases of the digestive system	1
Diseases of the genitourinary system	2
Diseases of the respiratory system	2
Diseases of the skin	1
Disorders of the nervous system	1
Endocrine, nutritional or metabolic diseases	2
Neoplasms (tumours)	8
Symptoms, signs or clinical findings, not elsewhere classified	4
Diseases of the musculoskeletal system or connective tissue	1

Abbreviation: *ICD‐11*, International Classification of Diseases, 11th Revision.

^a^ Participants' cultural identities were recorded based on self‐identification.

Three themes and an integrative theme were identified from the interview and diary data that captured parents' experiences of temporary tube feeding over time. These themes were as follows: (1) Parents were not prepared for tube feeding; (2) families experienced challenges managing their child's feeding tube; and (3) parents became self‐taught experts and needed to adapt to the changing demands of tube feeding. The need for ongoing support was identified as the integrative theme because it connected all aspects of parents' experiences throughout the tube feeding journey. Each of these themes remained consistent over time, although subcategories evolved as parents progressed through different phases of tube feeding. Theme categories, subcategories, and representative quotes are detailed in Supporting Information S1: Table S1.

### Theme 1: Parents were not prepared: “*We weren't really prepared for all the tube stuff*”

This theme comprises two main categories: informed decision‐making and knowledge. Within informed decision‐making, the categories and subcategories evolved across time, mirroring parents' progression from early uncertainty to growing awareness of their unpreparedness. Experiences shifted from initial uncertainty and limited involvement in decisions to recognition of knowledge gaps, culminating in retrospective reflections on improving preparedness.

#### Theme 1, category 1: Informed decision‐making

In the initial phase, parents consistently reported feeling underprepared for managing their child's feeding tube. Participants reported varying levels of involvement in decision‐making processes. Some described that decisions were “*made for us*” [P13], whereas others experienced more collaborative approaches in which decisions were “*really in consultation*” [P34]. Despite these differing experiences, most parents reported entering this process without an understanding of tube options, potential risks, or timelines, feeling uncertain and underinformed. As time progressed into the maintenance phase, parents reported greater involvement in decisions: “*the doctors . . . will tell [me] their plan . . . and see if I'm on board*” [P33]. Even with increased parent involvement, concerns about long‐term risks such as tube dependency persisted: “*no acknowledgement that long term tube feeding is what's happening, now 10 weeks, not the original plan‚ it was an acute issue . . . it should have been discussed”* [P16]. By the final phase, parents reflected critically on how the lack of early preparation and inclusion in decisions contributed to their prolonged uncertainty: “*we didn't actually have a time limit and so we didn't know when or if it [the tube] was gonna come out*” [P08].

#### Theme 1, category 2: Knowledge

Initially, parents reported receiving varying levels of information and education about their child's feeding tube. Whereas some found this helpful (“*the fantastic booklet that they gave us to go home with . . . had a lot of information*” [P20]), many felt overwhelmed by the timing and manner of delivery. Information was often provided at the point of discharge alongside many other activities, making it difficult for parents to absorb key details: “*you are so tired, especially on the final day when everyone is giving you information as you are leaving. When you get home, it's very difficult to recall those processes*” [P16]. Information gaps became increasingly evident during the maintenance phase, and many parents realized the education they received was inadequate: “*I wish we were told earlier about all the side effects of the tube, the rubbing, the sores it can cause in her nose and how the placement can make the [tube] aspirates difficult to do*” [P02]. This lack of practical knowledge left many feeling unprepared for complications at home. Some reflected that hospital education, although well intentioned, felt rushed and inadequate. One parent suggested a more structured approach for education would be valuable: “*There should be like a CPR course you need to do before you go home with a tube*” [P05].

In the final phase, parents reflected on what information they should have received from the beginning. They moved beyond recognizing the gaps in their care to specifically identifying the education, resources, and knowledge that would have made their experience less challenging. They emphasized the importance of timely, accessible education and training that evolved with their needs: “*knowing what the tubes were and more information about them at the time would have been good*” [P15]. Primary caregivers highlighted the need for clearer guidance for other family members, with one sharing about their partner, “*he feels like he doesn't know what questions to ask, and if they [clinicians] don't offer the information he doesn't know if he's missed something*” [P35]. By the end, parents recognized that well‐timed, comprehensive knowledge could have better equipped them for the challenges they faced throughout the tube feeding process.

### Theme 2: Families faced challenges managing their child's feeding tube: “*we are having a lot of trouble with the tube*”

Parents encountered various challenges throughout the tube feeding journey, categorized into: technical challenges and physical complications, equipment issues, and the broader impacts. Although many parents developed strategies to cope, they continued to face persistent obstacles throughout each phase of the feeding tube journey. The categories and subcategories show how parents' challenges evolved from initial technical difficulties to increasingly complex impacts on family dynamics and child development as tube feeding continued.

#### Theme 2, category 1: Technical challenges and physical complications

In the initial phase, parents reported that they struggled with basic tube management, particularly managing the side effects. Tape issues frequently caused irritation, rashes, and blistering, adding to children's discomfort. Aspirating the tube was often challenging: “*not being able to aspirate the tube and losing 30 min of our time . . . it's happened many times in a row and it's exhausting*” [P16]. Tube dislodgement was a constant worry, leading to frequent hospital visits for some parents: “*he's pulled the tube out twice since being home, we had to go to the hospital to get it put in*” [P13]. For many, tube reinsertions were not only time‐consuming but also a traumatic experience: “*he went floppy and the most white color and blue around his mouth‚ it was horrible*” [P25].

As children recovered and became increasingly active at home during the maintenance phase, parents faced intensified challenges managing the tube with their mobile children. Tube entanglement became a considerable risk: “*we wake up with the tube around his neck, it's quite concerning, so now we have to wake up to check on him*” [P21]. Even by the final phase, tube management remained demanding: “*it's a two‐person job . . . someone needs to hold him down and then the other person needs to place the tube*” [P17]. Parent‐reported impacts of the tube and tube feeding on their child, including both perceived benefits and challenges across the different stages of the tube feeding journey are detailed in Table S1. Although parents became better at anticipating tube challenges, these difficulties persisted over time.

#### Theme 2, category 2: Equipment challenges

Equipment‐related issues emerged immediately after discharge in the initial phase. Parents reported problems with durability, usability, and design of the tubes: “*The current tube we have is harder and the cap leaks constantly*” [P26]. Some noted that equipment was unsuitable for their child's needs: “*the feeding tube is designed for someone that can walk . . . not someone with the wheelchair*” [P01]. Ordering equipment proved particularly challenging, with frequent delays and errors (eg, “*finally the 3rd order was right, but this has been a lot of mucking about and hours on the phone*” [P15]). Regional families faced additional difficulties due to limited equipment access compared with participants who had access to the children's hospital. These issues persisted into the maintenance phase, with the logistics of storing supplies at home described as “*a nightmare*” [P20]. For some, the financial burden also became challenging over time: “*we wish we had been prepared for what was involved, the cost*” [P19]. By the final phase, parents emphasized the need for better‐designed, accessible, child‐friendly equipment and streamlined supply processes across healthcare services, with equitable access across geographical locations.

#### Theme 2, category 3: Multidimensional impact on child and family life

Initially, parents appreciated the health benefits of the feeding tube, such as improving their child's nutrition and energy levels. However, its impact on daily life, social interactions, and family dynamics soon became apparent. Many children faced restrictions in their activities: “*[Child] isn't happy with bolus feeds in the day‚ this means he's restricted*” [P35]. These limitations often left parents feeling isolated, compounding the emotional and overwhelming toll of caregiving for a child with a temporary feeding tube: “*I honestly don't know how long I'll be able to do this for*” [P04]. Family relationships became strained, with some partners hesitant to help primary caregivers (eg, “*my husband said, he'll stay away. . . . I think he's a bit scared about doing [a tube feed]*” [P23]).

In the maintenance phase, the emotional toll of tube feeding intensified as parents faced ongoing practical and psychological pressures. Tube management continued to be time‐consuming and stressful, and concerns about delayed child development surfaced—eg, “*[the tube is] starting to get in the way of her trying to crawl and walk*” [P27]. Social stigma added further anxiety: “*we don't go out with her, but when we do . . . the looks you get from the public . . . we don't like those looks*” [P19]. As these challenges continued, family routines became increasingly disrupted, and arranging school or daycare became a complex task that, for some, was “*a big project*” [P17]. By the final phase, long‐term psychological tube impacts were described: “*I firmly believe the feeding tube has created resentment of my daughter towards us as her parents*” [P02]. These impacts extended to sibling interactions, who were often restricted in their play to prevent tube‐related issues or dislodgements. Parents reported that most older children avoided public outings, whereas some younger children learned to associate certain rooms and routines with traumatic experiences like tube reinsertions. Parents mourned their traditional parenting role and emotional bond with their child, as medical caregiving became the primary focus. “*I lost part of being a mother and protecting him when he's scared [doing a tube insertion]*” [P14]. Ultimately, tube feeding disrupted a child's mobility, social life, and family dynamics.

### Theme 3: Parents developed resourcefulness: “*Knowledge is power*”

This theme comprises two main categories: parents figuring it out themselves and adapting to the tube. These categories show how parents progressed from problem‐solving to developing routines and expertise without formal support.

#### Theme 3, category 1: Figuring it out themselves

Throughout the entire tube feeding process, parents were compelled to develop their own strategies and solutions, often relying on improvisation, independent research, and informal networks to navigate the complexities of tube feeding management. In the initial phase, parents frequently described needing to be proactive and educate themselves about the tube while still in the hospital (“*I would just always make sure I was there [in ward rounds] and kept asking questions*” [P34]). Parents often created their own education materials through videos and photos to support their own learning. When their own efforts were insufficient, parents turned to their own networks for solutions. Friends, family, and private clinicians often filled in the gaps: “*my friend [a nurse] went through all the supplies with us, we didn't get taught what to do or what to use*” [P25]. Despite these efforts, knowledge gaps persisted, prompting parents to do their own research: “*I was looking up this . . . on a medical YouTube video . . . so that's how I learned about the feeding tube taping*” [P18].

During the maintenance phase, self‐reliance intensified as parents continued to encounter challenges requiring independent problem‐solving. Many described “trial‐and‐error” approaches to manage issues such as traveling and public outings. Parents had to be creative to find solutions for tube issues: “*we were doing lots of improvisations to get things to work*” [P01]. Through continued improvisation and problem‐solving, parents gradually developed the confidence and knowledge needed to advocate for their child's care: “*you speak up a bit more like once you've learnt and . . . you know what you're doing*” [P04]. Some figured out ways to assert their needs: “*when I put my foot down, suddenly then there was another option*” [P02]. With experience, parents became more adept at questioning conflicting clinical advice, which helped to prevent potential serious tube‐related errors: “*my husband and I were arguing about drawing the aspirate, we both got trained by different people . . . we brought him to the hospital, and the tube wasn't in the right place. Luckily I was there or Dad would have given him that feed*” [P13].

By the final phase, parents reflected on their self‐taught tube feeding journey, noting how they continued to learn independently and trusted their own judgment: “*it's just us, watching and learning, not being told much . . . we're just self‐educated mainly*” [P15]. Their overall experiences revealed a pattern of necessary resourcefulness, with some parents realizing they had greater confidence managing the tube at home, away from the scrutiny of hospital clinicians. Although parents demonstrated resourcefulness in developing their tube expertise, many emphasized that structured education and training would have significantly helped their journey and reduced unnecessary distress, noting they should not have needed to navigate the complexities of tube management themselves.

#### Theme 3, category 2: Adapting to the tube

In the initial phase, adapting to tube feeding involved considerable adjustments. Many parents felt overwhelmed but gradually developed routines for home, mealtimes, school, and outings through careful planning and organizational skills. Establishing these routines became essential to normalizing their lives with the feeding tube. For some, adaptation felt more like accepting an inevitable reality, “*resigned that it is what it is*” [P35]. During the maintenance phase, many parents were compelled to improve their child's quality of life by learning how to reinsert the tube themselves after distressing experiences in hospital emergencies (eg, “*she was screaming the entire time . . . it was overwhelming. . . . I know I can do better than that*” [P16]). These ongoing efforts to enhance home routines were driven by both the desire to improve their child's well‐being and the necessity of managing unpredictable situations effectively. Parents needed to adapt to unplanned logistical challenges and emergency situations: “*we had a power outage, and the [feeding] pump stopped working. We weren't ready, and it was chaotic*” [P20]. Equipment supply shortages further tested parents' adaptability, often forcing parents to reuse equipment despite the known risks: “*we definitely shouldn't be doing [it], but when you run out of [tube feeding] lines and there's no script, you don't have a choice*” [P27]. By the final phase, many had achieved a level of mastery in tube management: “*it [tube feeding] becomes pretty much second nature*” [P12]. Many parents reached a point of acceptance with the tube, recognizing it as an unavoidable aspect of their daily lives: “*we're just getting on with it*” [P17]. Although the process of adaptation was demanding, parents recognized the tube was a necessity for their child, “*better than having to stay in hospital*” [P31].

### Integrative theme: The critical need for ongoing support

**Figure 1:**
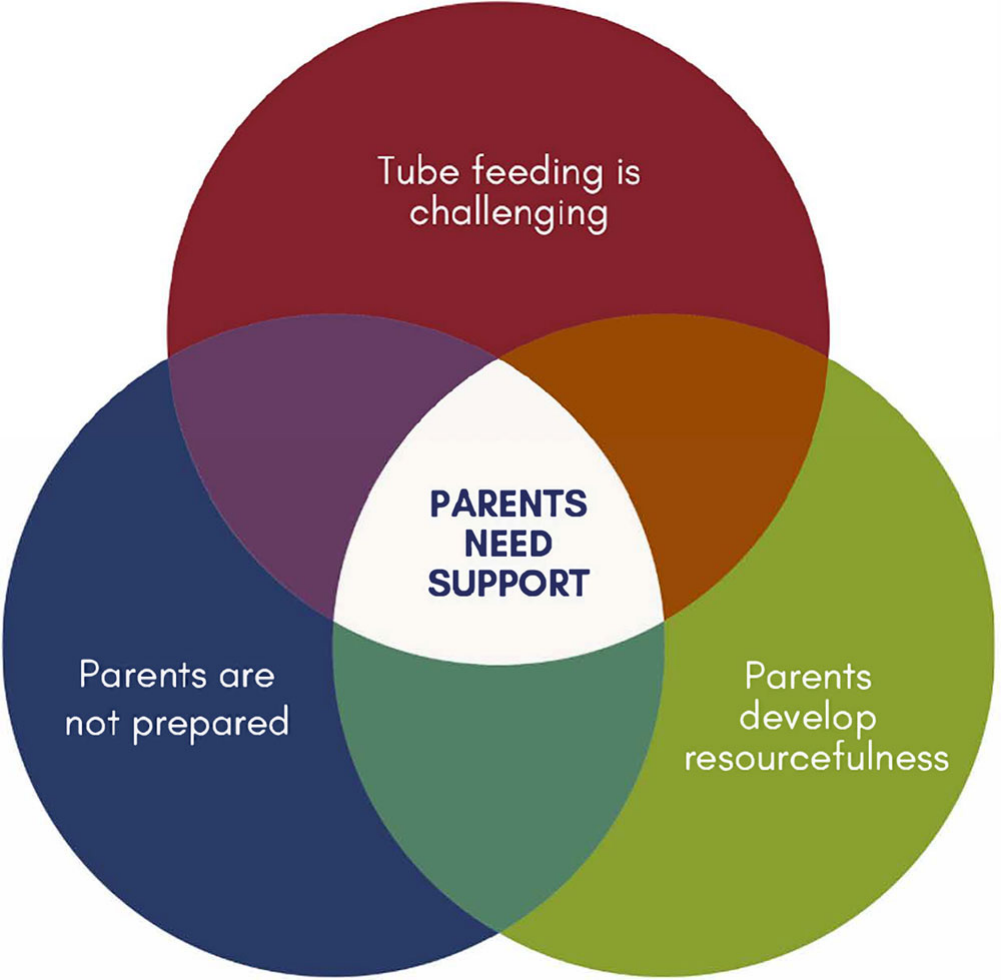
The integrative theme of parents needing support. This figure illustrates how parents' need for support emerges at the intersection of the three main themes: the difficulty of tube feeding, lack of preparation, and the necessity to develop resourcefulness.

The integrative theme describes parents' perceptions of how support systems shaped their experiences across their child's tube feeding journey, as described in Figure 1. Parents initially reported feeling overwhelmed by fragmented care, with many describing limited preparation and inconsistent guidance. As time passed, many parents reported that their support needs evolved from basic technical training to more complex psychosocial support as tube management became routine but social isolation increased. By the end, many parents reflected on how consistent, accessible support could have improved their entire experience, particularly for those facing cultural or geographic barriers.

#### Support gaps in preparation and education

The need for ongoing support was evident from discharge, with parents describing healthcare system gaps in tube feeding preparation, highlighting the need for individualized support systems.

Parents described receiving what they perceived as minimal feeding tube information about expectations, risks, and potential complications. These perceived gaps appeared to stem from what parents experienced as healthcare system deficiencies—“*the support once home with it is definitely lacking and it has made it harder to get*” [P27]—highlighting a desire for accessible and responsive support structures. Parents also described cultural and linguistic barriers as additional challenges. One parent from a culturally diverse background specifically identified the need for culturally appropriate support: “*I think the [feeding tube] information is a lot to take in . . . maybe we need an Indigenous officer there too, to like translate*” [P01]. Others expressed concern for those navigating these challenges: “*if I'm finding it overwhelming, then I can only imagine what . . . people from less privileged backgrounds than me or . . . that just aren't familiar with those [health] systems, they're going to struggle . . . if English is your second language*” [P16]. Finally, many parents reported feeling unprepared regarding tube dependency risks, perceiving this information was not adequately communicated to them.

#### Fragmented and inconsistent clinical support

Throughout their tube feeding journey, many parents described experiencing what they perceived as fragmented, inconsistent clinical care across several healthcare systems that affected their confidence and sense of support for the tube management. Some parents expressed feeling completely isolated: “*there's no one, we don't have anyone to ask*” [P19]. Other parents reported receiving contradictory clinical advice, which created confusion and uncertainty (eg, “*one nurse told us to check it [the aspirate] every time we do a tube feed, and another nurse told us we only need to check it once a day*” [P09]). These inconsistencies led parents to make their own judgments about critical aspects of tube management.

Parents described that the quality of support varied based on which clinicians they encountered: “*I've rang the children's ward . . . a couple of times and the ladies there are pretty good if you get the right ones*” [P05]. This perceived variability led parents to emphasize the importance of having “*a consistent team, everyone on the same page*” [P17], but such continuity of clinical support was seldom reported by parents. Even when parents did receive clinical input, many perceived it lacked substance and practical utility: “*they [clinicians] listened but weren't helpful*” [P02]. Parents described how they had to become mediators of what they perceived as conflicting clinical advice and navigate what they experienced as complex systems independently.

#### Barriers to accessing care

Parents reported experiencing significant geographic, cultural, and systemic barriers to accessing appropriate support and care. The perceived that the lack of accessible, community‐based support compounded challenges for many families. Many advocated for local health clinics to reduce travel burden, noting “*there are nurses available [in the health clinic who could do a tube insertion, and] . . . it would save so much time*” [P16]. Geographic disparities were particularly evident, with regional parents struggling for local follow‐up: “*we haven't actually seen the feeding team [in our regional hospital] . . . yet just [because] I think the appointments have been harder to get, they were pretty busy*” [P26]. Many parents reported feeling disconnected from healthcare services because of geographic and system barriers. One parent explained, “*We are still lost in the health care system. . . . Because we bounce between places, it depends which health network we are under, we haven't spoken to the home hospital*” [P19]. These reported barriers in care left families making critical care decisions without adequate guidance.

#### Development of alternative support systems

As parents perceived formal healthcare support to be inadequate or inconsistent, many reported developing alternative support systems to fill these gaps. Initially, many parents did not consider peer support necessary, believing their child's feeding tube would be a manageable short‐term measure. As the maintenance phase progressed, the tube feeding timeline extended, and complications manifested, online groups and social media emerged as essential resources. By the final phase, these platforms provided practical advice, emotional support, and connection with others facing similar experiences: “*I found out more stuff from this Facebook group I'm a member of than from the medical team*” [P17]. Throughout this evolution, family members and friends became critical components of many parents' makeshift support networks, offering practical assistance, knowledge, and emotional encouragement. However, this transfer of skills proved difficult for some: “*it's challenging teaching other family members [to use the tube]*” [P14].

#### Trust and relationships with clinicians

Parents described trust between themselves and clinicians as a crucial yet often fragile foundation for meaningful support. Parents' experiences varied widely, with some building strong, supportive relationships with clinicians, whereas others felt consistently dismissed or unheard. Sustained relationships with clinicians were perceived to provide continuity of support: “*we've managed to maintain the same pediatrician throughout . . . we don't have to keep explaining things to a new doctor*” [P03]. However, even with regular care, many parents still reported feeling isolated, highlighting the need for emotional support beyond technical tube management, with one parent wishing for “*just someone to talk to*” [P21]. Parents described encounters in which clinicians showed resistance to managing their child's tube reinsertion: “*it was like no one wanted to do it*” [P08]. Several regional families stated that “*we don't trust our local hospital*” [P19], highlighting the precarious nature of the parent‐provider relationship in the face of inadequate support.

## DISCUSSION

This study offers valuable insights into the experiences of parents managing temporary tube feeding at home. This is the first known longitudinal exploration of families' experiences with temporary feeding tubes, which highlights the multifaceted journey of families, revealing substantial gaps in preparation, persistent challenges, and the critical role of parents' resourcefulness. This study provides new insights by tracking evolving experiences of families over time, identifying unmet needs throughout all the phases of tube feeding. The findings underscore key areas for education, including tailored discharge education, ongoing follow‐up care, and culturally sensitive support systems.

### The preparation paradox

From the beginning, most parents felt ill‐equipped to manage their child's feeding tube. Parents frequently described being overwhelmed by the volume of information provided at discharge and noted that key details such as potential risks and the duration of tube feeding were inadequately addressed. This lack of clarity heightened parental stress and increased reliance on self‐directed learning. Research has demonstrated that without proper preparation, families are at greater risk of errors, potentially resulting in serious tube‐related consequences.^50^, ^51^ Whereas prior research noted similar issues regarding the lack of preparation,^27^, ^52^, ^53^, ^54^, ^55^ the longitudinal design of this study uniquely highlights the critical role of phased information delivery as a strategy to better prepare families throughout the tube feeding journey. Evidence from neonatal units^7^, ^56^, ^57^, ^58^ supports how phased ongoing information delivery enhances preparedness, boosts confidence, and reduces parental anxiety. Clinicians must see discharge as the beginning of care, not its conclusion, delivering ongoing support that empowers families throughout their tube feeding journey.

### The burden‐benefit balance

This study identified a complex tension between the therapeutic benefits of temporary feeding tubes and their substantial burdens on families. Tubes provide essential nutrition that supports weight gain and development, but they simultaneously create a cascade of challenges that evolve over time. It was observed that initial challenges for families were mainly practical (eg, aspirating the tube, taping, gagging from the tube); however, over time, these challenges grew into complex burdens, including developmental concerns, social stigma, strained parent‐child bonding, disrupted family dynamics, caregiver fatigue, and isolation. Although tube‐related side effects are well documented in literature^15^, ^16^ and known to clinicians,^52^, ^59^ this study reveals the impacts of these side effects often intensified over time, challenging the expectation that temporary feeding tubes would present only short‐term challenges. Additionally, families in regional and rural areas faced greater challenges due to the limited access to equipment, specialized care, and support, reemphasizing the urgent need for equitable healthcare services.^60^, ^61^

Age‐specific impacts created additional complexity in this burden‐benefit balance. Younger children faced mobility restrictions, such as limitations to crawling and rolling, whereas older children reported more tube‐related side effects such as discomfort from the tube in their nose and experienced greater social stigma and challenges attending school, similar to findings from other research.^62^, ^63^ These findings underscore the need for age‐specific interventions, such as providing specialized equipment or modifications to help younger children with mobility challenges and offering counseling or peer support for older children to manage social stigma and school‐related difficulties, thereby ensuring personalized holistic care.^64^

### Forced resourcefulness vs systematic support

A striking tension emerged between the remarkable resourcefulness demonstrated by parents and the perceived systemic failures that necessitated this resourcefulness. Parents transitioned from being novices to self‐taught experts over time, relying mainly on trial and error, personal networks, and informal peer supports. Parents without clinical guidance used peer support as a substitute. Although initially overlooked, peer support became indispensable for both practical advice and emotional connection. Online communities and informal networks have been shown to play a role in helping parents navigate their tube feeding journey.^65^, ^66^ However, this reliance on resourcefulness and peer support raises concerns about the accuracy and consistency of information, subjecting families to potential risks when formal support systems are not available.

Many parents reported feeling compelled to take on advocacy roles to secure appropriate care for their children, consistent with other research findings.^67^ Previous research shows mixed impacts: Whereas some parents found developing resourcefulness positive,^68^ others experienced serious psychological distress.^23^ Variability in clinician experience levels, as documented in previous research,^69^, ^70^ often led to inconsistent care, further increasing parental stress and uncertainty and ultimately forcing parents to build to work things out on their own. Relying on parental abilities, without adequate and consistent clinical support, is inequitable and risks compromising the well‐being of parents and their children. Addressing gaps in healthcare support is essential to reduce this responsibility parents carry.

### Caregiving role vs parenting identity

This study also revealed the long‐term emotional toll on primary caregivers, who often struggled to balance a nurturing relationship with their child while managing the demands of medical caregiving. The voice of fathers was noteworthy, with fathers participating in 8.1% of diary entries and 19.8% of interviews. This representation validates that tube feeding burdens impact all caregivers, demonstrating that these challenges transcend traditional caregiver roles. The exhaustion experienced by primary caregivers, often compounded by a lack of shared caregiving responsibilities (similar to findings from other research^71^), increases the risk of not adhering to tube‐related tasks correctly, and subsequent tube‐related errors.^51^, ^54^, ^72^ Similar to findings from Craig,^53^ many parents reported tube feeding strained the bond with their child, as tube‐related caregiving tasks took precedence over typical parenting roles. The persistent stress and sense of responsibility highlight the critical need to address the mental health of caregivers, an area that remains consistently undersupported.^18^ Notably, by the end of their tube feeding journey, parents demonstrated empathy toward other families, particularly those facing greater difficulties, reflecting the resilience they had developed through their experiences. These findings align with those from Tregay et al,^73^ who reported that two‐thirds of parents caring for children after major cardiac surgery found tube feeding to be the most stressful aspect of caregiving, overshadowing all other cardiac concerns. Interventions should focus on providing both practical support and mental health resources to help families navigate this challenging journey.

### Equity in access vs systemic disparities

The current study included participants from diverse backgrounds, with 12% identifying as First Nations and 18% as culturally and linguistically diverse. Families from these backgrounds reported additional challenges, such as language barriers, cultural misunderstandings, and limited access to appropriate resources, compounding the difficulties of tube feeding management. Participants highlighted the importance of involving cultural liaison officers, using interpreters, and providing personalized education to improve the care their child received. Healthcare systems must prioritize culturally safe practices, as described by Curtis et al,^74^ to ensure all families receive the support they need. By highlighting the experiences of diverse families, this study contributes new insights to the literature, providing actionable solutions for improving care and addressing inequities in healthcare delivery. Geographic disparities further exacerbated inequitable experiences, with regional and remote families experiencing additional barriers to accessing care, equipment supplies, and support services.^75^, ^76^ These findings align with established rural health inequities and emphasize the urgent need for telehealth expansion, improved coordination between metropolitan and regional healthcare systems, and recognition of how geographic isolation compounds existing social vulnerabilities.

### Trust dynamics in healthcare relationships

Trust and confidence in the healthcare system appeared as an important factor in parents' experiences, with many families reporting a loss of confidence because of the perception of inconsistent or insufficient clinical support and unclear communication. This left some families in this study feeling isolated and unsupported, increasing the risk of inadequate healthcare input for the child's tube feeding management. This study found that trust was particularly eroded by inconsistent advice, with parents reporting contradictory information from different clinicians about fundamental care procedures. Trust is crucial in healthcare, and a meta‐analysis^77^ has reported that those who trusted their clinical team experienced better health outcomes, higher quality of life, and greater satisfaction with their care.^77^ Healthcare system distrust may prevent individuals from seeking timely care, following clinical recommendations, or attending follow‐up appointments, potentially compromising their health outcomes.^78^, ^79^ Building trust throughout the entire duration of tube feeding is essential and could be achieved by involving families in decision‐making processes and providing consistent, appropriate support. Trust is cultivated when healthcare teams include, acknowledge, and actively involve parents in their child's tube feeding care.^22^

### Limitations and future directions

This study provided important insights into parents' experiences of temporary tube feeding over time, but some limitations are acknowledged. Several participants were lost to follow‐up despite retention efforts, suggesting that families experiencing the greatest challenges with temporary tube feeding may be underrepresented in the findings. A limitation of this study was that participants came from a single quaternary children's hospital, although many children received follow‐up care in diverse regional settings. Child participation was minimal, underscoring the need for age‐appropriate, play‐based data collection methods in future to capture their perspective.^80^ Future studies could explore the long‐term psychosocial impacts as children transition from tube feeding, investigate the experiences of diverse family members, and examine healthcare disparities in culturally diverse and rural populations. This study focused exclusively on parents' experiences. Future research should incorporate healthcare professionals' perspectives managing children with temporary feeding tubes. Additionally, quantitative measures of economic impact, caregiver burden, and children's quality of life would complement these qualitative findings.

## CONCLUSIONS

This study reveals parents' struggles with persistent uncertainty, multifaceted burdens, and significant unmet support needs throughout the temporary tube feeding journey. Parents described challenges navigating complex healthcare systems, managing technical aspects of tube care, and coping with emotional impacts while attempting to maintain family normalcy. Cultural and geographic factors further complicated these experiences, with diverse families reporting communication barriers and rural families facing additional access challenges. These findings highlight the urgent need for systemic improvements in pediatric temporary tube feeding support, including standardized education and improved discharge processes, that address the lived experiences these families. Healthcare systems can better support families by responding directly to these identified needs.

## AUTHOR CONTRIBUTIONS

Claire Reilly, Rebecca Packer, Jeanne Marshall, and Nikhil Thapar contributed to the conceptualization and design of the study. Claire Reilly contributed to data collection, data curation, and formal analysis, with Rebecca Packer contributing to data analysis. Claire Reilly prepared the original draft. Claire Reilly, Jasmine Foley, Rebecca Packer, Jeanne Marshall, and Nikhil Thapar critically revised the manuscript. All authors agree to be fully accountable for ensuring the integrity and accuracy of the work and have read and approved the final manuscript.

## CONFLICT OF INTEREST STATEMENT

None declared.

## ETHICS STATEMENT

Informed written consent was obtained from all participants. The sample size was determined by including all eligible participants who could be recruited within a defined time period from August to December 2023.

## Supporting information

Supplementary Information.
