# Supplementary material for: “It's just us”: Families' experiences with temporary tube feeding
Source: Nutr Clin Pract. 2025 Aug 20;41(2):647–61. doi: 10.1002/ncp.70019 (PMC12982660; doi:10.1002/ncp.70019)
Supplement: Supplementary file 1 — Supplementary Information. [file NCP-41-647-s001.pdf]

File S1. Diary Template

This diary template was provided to parents and caregivers to document their experiences managing a child's temporary feeding tube. The diary included both structured questions and open-ended reflections to capture daily routines, emotional responses, healthcare interactions, and significant events.

Section 1: Managing the Feeding Tube

Who is completing this diary?

Child

Mother

Father

Other family member/caregiver (please specify)

Please rate the following on a scale of 1 (terrible) to 5 (great):

How are you managing the feeding tube? (e.g., using the tube, replacing the tape)

How is your family managing with the feeding tube?

How are you feeling about the feeding tube?

How is your family feeling about the feeding tube?

Section 2: Daily Routines

In the past week, how well have the following activities been going? (1 = not well at all, 5 = extremely well)

Going to school/daycare

Meal times

Going out in public (e.g., shopping, park)

Attending events (e.g., birthdays, family dinners)

Going on holidays

Section 3: Time Spent on Tube Feeding

On average, how many hours per day does each of the following take? (Select 0–9 hours)

Giving a tube feed

Preparing the feeding tube (e.g., syringe setup, pH strip, feeding pump)

Replacing the tape to secure the feeding tube

Replacing the feeding tube

Please share if anything was different at home with the feeding tube in the last week (e.g., tube needed replacing multiple times):

Section 4: Healthcare Interactions

Have you had any allied health reviews about the feeding tube? (Tick all that apply)

Dietitian

Speech Pathologist

Occupational Therapist

Feeding Therapy Team

Other (please specify)

Did you have a recent hospital appointment about the feeding tube? Yes/No

<u>If yes, who did you see? (Tick all that apply)</u>

Doctor

Dietitian

Feeding Therapy Team

Nurse

Speech Pathologist

Occupational Therapist

Other (please specify)

What information were you given about the feeding tube during the appointment?

<u>Please indicate your agreement with the following statements (Strongly agree to Strongly disagree):</u>

At the hospital appointment, I felt listened to.

At the hospital appointment, I felt supported.

At the hospital appointment, my questions were answered.

At the hospital appointment, my needs were met.

At the hospital appointment, I was given a plan for managing the feeding tube.

Is there anything else from the appointment you'd like to share?

Section 5: Reflections and Experiences

Is there any information you wish you had been told earlier about the feeding tube?

What has been your worst experience related to the feeding tube so far?

What has been your best experience related to the feeding tube so far?

Is there anything else you would like to share about your experience of living with the feeding tube?

Figure S1: Participant Recruitment, Retention, and Interview Completion Across Phases of Tube Feeding

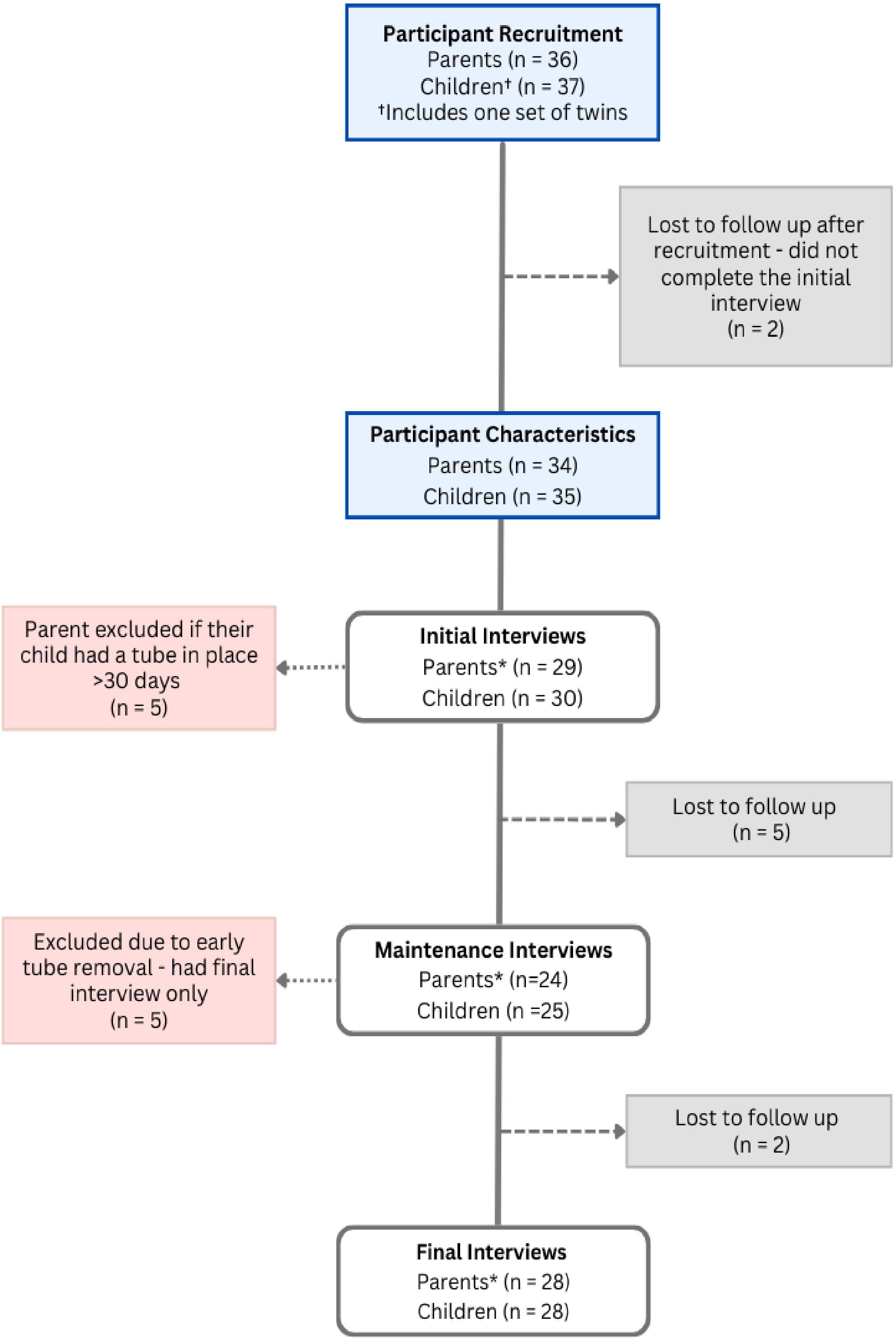

Note. *One parent completed interviews on behalf of the twins. At the first two timepoints, this was treated as a single interview, in line with the parent's preference, as both children had similar experiences. For the final interview, one twin's tube had been removed, so separate interviews were completed.

File S2. Parent Interview Guide

These interview guides were used at three key timepoints (Initial, Maintenance, and Final) to explore parents' experiences of managing temporary feeding tubes in children across the tube feeding journey.

Initial Interview Guide (Tube Insertion Phase)

1. When was the tube inserted?
2. What reason were you given for your child needing a feeding tube?
3. Who explained this to you (e.g., doctor, dietitian)?
4. Were you told in advance that your child would need a tube?
5. Were you involved in the decision to insert the feeding tube?
6. Who else was involved in the decision?
7. Were you involved in planning the feeding regimen?
8. Did you feel this was a joint decision or one made for you?
9. What is your understanding of how long your child will need the feeding tube?
10. Were you informed of any risks or benefits related to the feeding tube?
11. Were any alternatives to a feeding tube discussed?
12. Were you told about different types of feeding tubes?
13. Were you provided with information about the feeding tube? If yes, what was it (e.g., booklet, website)?
14. What was helpful about the information provided?
15. What could have been improved in the information provided?
16. Were you referred to anyone to support your child’s eating or drinking?
17. When you go home, where do you think you will get information or advice about the feeding tube?
18. Would you like to speak to other families who have a child with a feeding tube?
19. Who do you think is the main person making decisions about your child’s feeding tube?
20. What are the most important things for you and your child in this situation?
21. Who do you feel you can go to for support regarding the feeding tube?
22. Were you given any education about managing the feeding tube at home?
23. Were you taught how to insert the feeding tube?
24. Have you considered the cost of the feeding tube? Was this explained to you?
25. Do you have any other thoughts about the feeding tube you’d like to share?

Maintenance Interview Guide

1. Tell me about the experiences you are having with the feeding tube.

2. What is the impact of your child having a feeding tube?

3. Do you have any feedback on the care you have received regarding the feeding tube?

4. Did you feel able to talk to hospital clinicians about the feeding tube?

5. How was the communication?

6. Did you feel respected?

7. Did you feel your needs were met?

8. Was there any information you wish you had been told earlier?

9. What could be improved?

10. What support systems or networks do you have to help with the feeding tube?

11. Where are you getting information about the feeding tube?

12. What is your understanding of how long your child will need the feeding tube?

13. Have you been told about any side effects of the feeding tube?

14. Who do you think is the main person making decisions about your child's feeding tube?

15. What are the important things for you and your child in this situation?

16. How has tube feeding affected your relationship with your child?

17. Are you receiving any feeding therapy for your child's oral intake?

18. Do you have any other thoughts about the feeding tube you'd like to share?

Final Interview Guide (Tube Removal / End Phase)

1. Tell me about your overall experiences with the feeding tube.

2. What was the impact of your child having a feeding tube?

3. Do you have any feedback on the care your child received?

4. Did you feel able to talk to hospital clinicians about the feeding tube?

5. How was the communication?

6. Did you feel respected?

7. Did you feel your needs were met?

8. What information were you given about the tube?

9. What could be improved?

10. How did you troubleshoot issues with the feeding tube?

11. What support systems or networks did you have to help with the feeding tube?

12. Where did you get information about the feeding tube?

13. What was your understanding of the duration of tube feeding?

14. Who was the main person making decisions about your child’s feeding tube?

15. Has tube feeding affected your relationship with your child?

16. What were the most important things for you and your child in this situation?

17. Was there any information you wish you had known earlier about tube feeding, and why?

18. What did you think of the financial cost of tube feeding?

19. Do you still have follow-up at the hospital? If yes, who are you seeing?

20. Did the feeding tube lessen or increase your worries about your child?

21. What was the hardest part of caring for your child with a feeding tube?

22. If you could change one thing to improve the healthcare system for children with feeding tubes, what would it be?

23. How confident did you feel navigating the healthcare system with your child’s feeding tube?

24. What were your thoughts when the tube was removed? *(do not ask if the tube remains in place)*

25. Do you have any other thoughts about the feeding tube you’d like to share?

Table S1. Thematic Categories, Subcategories, and Representative Quotes

Themes 1 - 3 and the Integrative Theme are presented below.

Theme 1: Parents were Not Prepared for Tube Feeding

Categories	Subcategories	Example participant quotes		
		Initial	Maintenance	Final
Informed decision making	Decision to insert the feeding tube	“yeah, I think so I didn't feel like it was thrust upon us, but we knew that it was recommended ...but it didn't feel like it we couldn't say no” [P16]<br><br>“I don't really know anything about any other feeding tube just this one” [P23]	“it's me 100% me [making decisions about the tube]” [P17]	“they just said they were gonna put a new tube in when her NG came out, they didn't tell us it was TPT...I didn’t know what the TPT was...” [P18]
	Reason for tube feeding	“[told the reason for the tube was] just because their swallowing muscles were weak” [P37]	“they put him on the NG for weight, but it's actually a whole other reason as to why it's weight is affected” [P14]	“we've never really found out why.. conclusively why we need the tube” [P21]
	Risks discussed	“no, we haven't really been spoken to about risks per say, I know the benefits and we're getting nutrition into her’ [P36]	“"...some nurses had mentioned that if we did [tube insertion] ourselves…they stop breathing or there's some sort of valve or something that we can damage in their throat” [P26]	“so there was never this.. formal introduction to.. your child is on the tube, here's the risks, here’s who you’re going to be talking to, here’s what to do for management of it” [P16]
	Duration of tube feeding	“[I was told that child] just [needs the tube] until they're on full suck feeds” [P04]	“No one has talked about how long the tube needs to stay in for” [P01]	“We weren't told a time frame but initially we thought a couple of weeks” [P03]

Knowledge	Information	“One thing to make it better would be if there was more readily available information about what it’s like at home with other kids around” [P32]	“[Wish we had] more information about people’s experiences at home” [P24]	“I think knowing what the tubes were and more information about them at the time would have been good” [P15]
	Education	“You think you can learn how to use the tube and then you go home but it's only shown once” [P25]	“we want to know more about starting her with solids, but we are worried with the sticky foods like rice could get stuck around her tube and we don't know how to manage that” [P26]	“when I took her to ...the [speech pathologist]… when they undressed her and redressed her, they put the tube under the clothes, and that was so useful to know, to help keep it in I didn’t know that, it would have been useful to know” [P04]

Theme 2. Tube Feeding is Challenging

Categories	Subcategories	Example participant quotes		
		Initial	Maintenance	Final
Technical Challenges and Physical Complications	Tube management	"Everything is difficult, getting them into the car, even in their capsules it's hard to set them all up so [the tube is] not in their face" [P15]	"The dangliness of the tube gets frustrating" [P09]	"I found it hard to hang the bottle in the car… I use [the] little coat hanger hook that is on most cars, but every time we're in the corner, it fell off" [P30]
	Tube position	"I don't want the tube [to]... move up or go somewhere it shouldn't go" [regarding the risk of the tube position not being in the stomach] [P29]	"we struggle very much with trying to aspirate, and quite often when we tried it at home she'd end up spewing when we're doing it" [P02]	"you [have] got to be very cautious as to [the] position of [the nasojejunal tube], because if I mean [the child] coughed and sneezed one time, and it went back up and then and kinked [and needed to be replaced]" [P18]
	Tube taping	"Tape changes are difficult, he doesn't want to do it... he doesn't like the smell of the skin protective wipes, his anxiety" [P35]	"the tape has been the hardest thing to manage" [P03]	"definitely the hardest part… the tape change so that she doesn't pull out her [tube]" [P24]
	Getting tangled in the tube	"[child] plays with it [the tube], getting it stuck in her toes, wrapped around her arms and neck" [P19]	"we wake up with the tube around his neck, it's quite concerning, so now we have to wake up to check on him. We hear him moving and we wake up. And he can't sleep in another	"She gets tangled quite easily [in the tube]" [P06]

			room, because it's not safe for him" [P21]	
	Mobility	"Maybe if they were to make the tube a bit longer he might be more mobile with the tube" [P09]	"It's a lot more difficult when you've got a toddler running around wanting to do stuff, and yet you've gotta try and have him hooked up" [to the feeding pump]" [P15]	"they start to move around and roll you're worried it's [the tube is] gonna get wrapped around [their] neck" [P05]
	Pulling out the tube	"He's pulled the tube out twice since being home, we had to go to the hospital to get it put in and then he had a seizure in the car on the way home and pulled it out again" [P13]	"it's so much easier for her to pull it out now and I have to put it back in more often and upset her more" [P06]	"Well [the hardest thing] was him constantly pulling it out" [P29]
	Tube re-insertions	""Having to put the tube back in and worry that it'll be in her lung" [P04]	"learning how to do the tube insertion has been incredible" [P21]	"it can be tedious just swapping the actual tubes over" [P09]
Equipment challenges	Durability, usability and design of the equipment	"The current tube we have is harder and the cap leaks constantly" [P26]	"Changed the tube multiple times after it being pulled out. Ran out of supplies" [P19]	"it would have just been nice if if a hospital pole would have been put, you know, like a consideration to offer to us, would have been really helpful for that transition home" [P10]
	Equipment ordering	"Also…regarding the feeds have been appalling to deal with. Takes forever to get through. My first order went to Western Australia" [P15]	"dealing with a third party for her [tube] supply... 2 hours on hold waiting [on the phone] " [P27]	"it's just a bit annoying that you have to order the parts for the pump from one place and then and get the other stuff for the pump, like the the nasal tube itself and all the other stuff from

				a different place I mean, it's like, can't you just order it all from the same place wouldn't it be easier?" [P29]
	Cost	"Expensive to purchase the pole [for the feeding pump] - $390" [P20]	"Financially the feeding tube is becoming difficult as I cannot work with her having NG feeds' [P20]	"I guess for long term [buying tube supplies] could be like expensive for people" [P30]
Multidimensional Impact on Child and Family life	Benefits	'My baby is putting on weight which is a result of her getting enough nutrition" [P24]	"She is gaining weight on the continuous feeds' [P18]	'he … gained weight consistently' [P14]
	Developmental concerns	-	"we're worried now that [the tube is] gonna impact her ability to begin solids … because she's losing all of these crucial reflexes that are required steps" [P16]	"if we don't learn to feed, do we not learn to do other things?" [P24].
	Impact on the child	"he doesn't mind the tube but doesn't let us do the feeds, he's self conscious about feeds going in. Just refuses. He tolerates the night time feed" [P35]	"she hates [the tube] now. She's started to run away when she knows that she needs to have her meds or a feed... we can only hook her up to her feeds now when she is asleep" [P02]	"We missed out on many things. We wanted to take him to the free swimming lessons but they only ran up to 5 months and therefore [my child] wasn't able to take part" [P03]
	Impact on the wider family	"my husband isn't as vigilant with flushing the tube …. [he] had to sleep with him [child] last night and then got frustrated with the alarm beeping with the occlusion	"Upcoming [family] holiday plans needed to be cancelled due to the tube and feeding" [P20]	"I found grandparents/carers were not confident with the tube and therefore we were not able to leave our child with anyone so we could be at work etc" [P13]

		so he [husband] just turned it off" [P35]		
	Daily life burdens	"I haven't gone out by myself around tube feeding, there is just a burden of bringing and organising everything, packing the machine, extra cord, everything" [P25]	"She still doesn't enjoy [the tube], she wants to be free of It" [P01]	"the hardest part was when we first got home I was a little bit anxious just to replacing and doing everything on my own for the first time without the nurses to back me up" [P34]
	Emotional impact on primary caregiver	"Some days I feel overwhelmed with the feeding tube, making sure that it doesn't get caught on anything or [child] doesn't rip/pull at it" [P33]	"Going home [with the tube] was scary P10	"it's upsetting when you have this very smiling, trusting, happy little face, looking up at you with so much love, and you have to do something [a tube insertion] that's just gonna really upset them" [P16]
	Tube impacts on bonding	-	"Its just different, it's just it's not as personal as bottle feeding" [P19]	"Because…you don't get to hold them and feed them bottles ...so I didn't really get any like connection time with her ... at all really so... they're just kind of these little humans that lay in the cot and you just look at them and change their nappies" [P04]
	Medical caregiving	-	-	"It's just hard from a Mum point of view, having your child screaming at you because you've gotta do something for their health" [P06]

Impacts of the Tube and Tube feeding on a Child (Pertaining to Theme 2)

Categories	Subcategories	Example participant quotes		
		Initial	Maintenance	Final
Side effects of the tube and tube feeding on the child	Weight gain	“My baby is putting on weight which is a result of her getting enough nutrition” [P24]	“She is gaining weight on the continuous feeds” [P18]	“he would gained weight consistently” [P14]
	Nutrition	“to make sure it gets the right nutrition” [P07]	“I know it's giving him all the nutrients that he needs” [P10]	“because I knew that he was getting what he needed” [P30]
	Energy	“so that she can fight many infections that she's been getting and have more energy” [P06]	“having a lot more energy” [P20]	“he's conserving his energy and hopefully will catch up growth wise, he’s got energy now it's like he's been able to really develop” [P17]
	Reflux	“Baby has developed terrible reflux and we are now having to hold her up for an hour after feed” [P26]	“she spews a lot and has a lot of reflux like they did say that [the tube] could have been part of the reason” [P05]	“when she has a bit of reflux and then she vomits, the tube is irritating” [P24]
	Gagging	“The feeding tube causes [child] to gag at lot” [P01]	“if she gags and she can't kind of slow herself down, I think having the tube down makes it harder for her to control” [P24]	“he hated the feeling … that liquid [was] going down the tube down the back of his neck and his gag reflex was just so sensitive” [P10]
	Vomiting	“trying to do aspirates with the syringe it gets stuck when doing it - makes her vomit - very difficult” [P02]	“it [the tube] contributes to some of his vomiting” [P14]	“the biggest issue was like adapting to his vomiting” [P21]
	Irritation	“unsure if the tube causes a strange sensation when the child is swallowing food” [P13]	“[the tube] irritates her throat” [P01]	“I think the feeling she's had enough of the feeling of it [the tube] in her throat, I think and just wanted to

			swallow things without it being in her throat" [P31]
Weight loss	"He's supposed to gain weight with the tube, but he's losing weight" [P15]	"the tube wean with his weight loss was hard so I'm thankful that he has [the tube] it" [P17]	
Vagal response [risk of tube insertion]	"[child] having a vagal response to it [the tube] being put down" [P04]	-	-
Tube tape	"keeping it attached to his face, the tape, it's always coming off, staying on is the hardest … [we've] tried everything" [P30]	"when the tape comes loose … she kinda gets her little fingers underneath [the tube] and pulls it half out" [P12]	"[the hardest part of managing the feeding tube was] just the maintenance of [the tape], so making sure that the sticker [tape] got changed at the right intervals" [P35]

Theme 3: Developing Resourcefulness

Categories	Subcategories	Example participant quotes		
		Initial	Maintenance	Final
Figuring it out themselves	Self-education in hospital	“I know her dad even recorded the first tube feed that the nurses did so we've got it on record as well” [P22]	“I think it makes sense to teach tube insertions in emergency, I think it should be shown, parents do it under observation of nurse supervision there in emergency like on site training with a few tries” [P21]	“But now being at home and being able to do it by myself, it's a lot easier cause I don't feel … the stress of someone watching me, making sure that I'm doing it right” [P06]
	Independent learning	“I’ll probably research stuff online” [P36]	“We've been troubleshooting ourselves because we don't know who to contact... we read a lot and took what we feel might work.” [P19]	“I did I did admittedly, google any protocols for the tube removal” [P34]
	Using external networks	“We've got a lot of medical people in our family so I think we just kind of talked to them about it and had a basic understanding” [P24]	“we do have a private Paediatrician that we do pay for.. so she has helped us quite a bit” [P26]	“[asked a] friend [a radiologist] coming to help us put it [feeding tube] down” [P21]
	Problem solving	“we use water flushes to check [tube] placement, it feels absolutely wrong but we aren’t in a position to do otherwise.”	“[I] think it would be quite stressful if I wasn't in that [health] industry and didn't have that background” [P20]	“finding out about the waterproof tape … has been a bit of a game changer because … it would have saved us doing tape

		[P16]		changes" [P27]
	Advocating	-	"If a doctor or a nurse puts [a tube in] …and you don't think it was measured right or you don't think that the pH [measurement] they got was good enough... just say something, make sure that it's 100%... always trust your gut with it." [P05]	"definitely as we went along I think I got more confident in asking for things, but I don't think that's a good thing … I don't think it was great to have to learn how to do that or … build up that confidence I think that comes from not feeling…overly confident with the mechanisms of [the hospital system] from the very beginning" [P16]
Adapting to the tube	Acceptance and routine	"The tube is getting easier to use the longer we have it" [P20]	"we've had it so long now we've gotta get used to it" [P08]	"I've learned a lot more about it and I feel more confident now" [P22]
	Skill and mastery	"Getting much better with managing the feeding tube where it doesn't take that long to insert, flush, test and tape" [P33]	"It's getting easier, it's a mix of talking to the nurses, watching the nurses and the longer you do it the easier it is, kind of tweaking things" [P02]	"I think we are experts now" [P15]

Integrative Theme: The Need for Ongoing Support

Categories	Subcategories	Example participant quotes		
		Initial	Maintenance	Final
Barriers to care	Lack of ongoing care	“There were two times we asked for help about the aspirating concern, the doctor and [name of the ward] and neither could help. This is the main issue” [P02]	"No updates from any teams regarding future plans for the feeds" [P20]	“I feel like they've kind of dropped us off a bit …but I carried through with the plan as I was going to do it and no one from the hospital has contacted us in a few months now” [P35]
	Inconsistent advice	“One nurse made the assumption that [the feeding tube] would be a lifelong prospect and made a statement that we might have to accept that our child will never eat food again. That was upsetting and probably out of her scope of practice, however we felt confident that we could seek clarification from the doctors” [P13]	“I think definitely there's, like, maybe a universal standard for kids …like amongst the hospitals about the equipment that's used, and we have had different nurses tell us…she's got the wrong size tube in…it is quite confusing because everyone has their own opinion on it” [P26]	“it seems like none of the specialties talk to each other, and then we do a lot with our GP, who then doesn't get any feedback from anyone” [P19]
	Lack of parental involvement	-	-	“sometimes we weren't we felt we felt like we weren't listened to” [P08]

Support	Hospital is a support	“They said that there's someone [on the ward] that we can come to if we need stock… and ED if we need help to put the tube in” [P07]	“nurse and doctors are very knowledgeable and clear” [P12]	“I do also have a lot of help from the [hospital] team." [P06]
	Peer support systems	“like a Facebook group or something like that where you can sort of pop on and say, hey, I’ve got this issue or what do you recommend for this and … go from there” [P20]	“I have a really close friend who her son's now on a PEG [feeding tube] so she's given me lots of information” [P17]	“connecting with these other mums and hearing how they managed things [feeding tube related]… has given me a bit more confidence around the tube” [P24]
	Trust	“I wouldn't ask her [private clinician] I’d be asking someone from the hospital [about the feeding tube]” [P16]	“we've managed to maintain the same Paediatrician throughout, so she’s very invested and knows everything from the case, obviously we don't have to keep explaining things to a new doctor and so that's been really good” [P03]	“I feel I feel like I trust them enough” [P22]
	Empathy for other parents	-	-	“I’ve often wondered…people in rural areas, how do they get by cos I’ve made so many trips to the hospital” [P17]

	“and you have more like respect for people and parents… they go through stuff like this [having a child with a feeding tube] cause this is it is a bit harder” [P29]
